# Unsupervised Monocular Depth Estimation Method Based on Uncertainty Analysis and Retinex Algorithm

**DOI:** 10.3390/s20185389

**Published:** 2020-09-21

**Authors:** Chuanxue Song, Chunyang Qi, Shixin Song, Feng Xiao

**Affiliations:** 1 College of Automotive Engineering, Jilin University, Changchun 130022, China; scx@jlu.edu.cn (C.S.); qicy17@mails.jlu.edu.cn (C.Q.); 2 School of Mechanical and Aerospace Engineering, Jilin University, Changchun 130022, China; 3 State Key Laboratory of Automotive Simulation and Control, Jilin University, Changchun 130022, China; xiaofengjl@jlu.edu.cn

**Keywords:** monocular depth estimation, Retinex algorithm, uncertainty analysis

## Abstract

Depth estimation of a single image presents a classic problem for computer vision, and is important for the 3D reconstruction of scenes, augmented reality, and object detection. At present, most researchers are beginning to focus on unsupervised monocular depth estimation. This paper proposes solutions to the current depth estimation problem. These solutions include a monocular depth estimation method based on uncertainty analysis, which solves the problem in which a neural network has strong expressive ability but cannot evaluate the reliability of an output result. In addition, this paper proposes a photometric loss function based on the Retinex algorithm, which solves the problem of pulling around pixels due to the presence of moving objects. We objectively compare our method to current mainstream monocular depth estimation methods and obtain satisfactory results.

## 1. Introduction

In contemporary research, the methods of monocular depth estimation based on deep learning are divided into the following six types: supervised, unsupervised, semi-supervised, conditional random field (CRF), joint semantic segmentation, and information-assisted depth estimation. In practical applications, the six methods overlap each other, and there are no strict boundaries. 

When monocular vision research emerged, many scholars trained neural networks in a supervised manner. In 2014, Eigen et al. [1] used deep neural networks for monocular depth estimation for the first time. They proposed the use of neural networks of two different scales to estimate the depth of a single picture. The coarse-scale network predicted the global depth of an image, and the fine-scale network optimized local details. In 2015, Eigen and Fergus et al. [2] proposed a unified multi-scale network framework based on the aforementioned work, and used it for depth prediction, surface normal vector estimation, and semantic segmentation. Liu et al. [3] combined a deep convolutional neural network with a conditional random field to propose a deep convolutional neural field to estimate the depth of a single image. Based on the work of Trigueiros et al. [4], Liu et al. [5] proposed a comparative study of four classification algorithms for static hand gesture classification using two different hand features data sets. Li et al. [6] proposed a multi-scale depth estimation method: First, a deep neural network was used to regress the depth of the super-pixel scale, and then multi-level conditional random field post-processing was used to optimize the combination of the super-pixel scale. Laina et al. [7] proposed a fully convolutional network architecture based on residual learning for monocular depth estimation. The network structure is deeper and does not require post-processing. Cao et al. [8] treated the depth estimation problem as a pixel-level classification problem. 

Conditional random fields (CRFs) have always performed well in the field of image semantic segmentation. Considering the continuity of depth values, researchers have begun to apply CRFs to solve depth estimation problems and have achieved some results in recent years [9]. In addition, some researchers [10,11,12,13] combined semantic segmentation with depth estimation. They used the similarities between depth and semantic information to make the two complement each other to achieve the goal of improving accuracy.

Due to the particularity of monocular depth estimation, the supervised training of neural networks is often limited by the scene. Thus, to overcome the need for ground truth data, unsupervised training of a network is a popular research topic. The basic idea is to use either left and right images or inter-frame images, in combination with epipolar geometry and automatic encoders to solve the depth. Many scholars have begun to study the monocular depth estimation of unsupervised learning. Zhou et al. [14] proposed a method that uses a sequence of images taken by a monocular camera as a training set and uses an unsupervised method to train a neural network for monocular depth estimation. Yin et al. [15] improved upon the aforementioned methods by adding a part to estimate the optical flow, extracting the geometric relationship in the prediction of each module, merging them for image reconstruction, and integrating depth, camera motion, and optical flow information for joint estimation. Reza et al. [16] proposed an unsupervised learning monocular image depth and motion estimation method using 3D geometric constraints. Clement et al. [17] used image reconstruction loss to train the network, and output the disparity map through the neural network. Zhang et al. [18] solved the problem of scale uncertainty in unsupervised learning using binocular data to jointly train depth estimation and visual odometer networks. Garg et al. [19] proposed the use of stereo image pairs to achieve unsupervised monocular depth estimation without the need for depth labels, similar to that of automatic encoders. Godard et al. [20] further improved upon the above method, using the consistency of the left and right images to achieve unsupervised depth prediction. Kuznietsov et al. [21] proposed a combination of supervised learning methods labeled with sparse depth maps and unsupervised learning methods, namely semi-supervised learning, to further improve performance. 

The current unsupervised monocular depth estimation studies used similar pixel value subtraction methods (some researchers also used the SSIM algorithm) in terms of photometric loss. The SSIM algorithm is shown in Equation (1)

(1) $$SSIM\left(x,y\right)=\frac{\left(2\mu_{x}\mu_{y}+C_{1}\right)\left(2\sigma_{x}\sigma_{y}+C_{2}\right)}{\left(\mu_{x}^{2}+\mu_{y}^{2}+C_{1}\right)(\sigma_{x}^{2}+\sigma_{y}^{2}+C_{2})},$$

where *x* and *y* represent two images to be compared, $C_{1}$ and $C_{2}$ represent constants, μ represents the average gray level, and σ represents the structural similarity of the image.

We think that photometric loss primarily affects the depth of the edge of the object in the image, which leads to an unclear depth map around the contour of the object. In addition, the current convolutional neural network used for monocular depth estimation has strong expressive ability, but it cannot evaluate the reliability of the output result. In this study, the variance of the training neural network was used to construct the uncertainty loss function equation. Uncertainty estimation has a long history in neural networks as well, starting with Bayesian neural networks. Different models are sampled from the distribution weights to estimate the mean and variance. This method is simple and effective. Many scholars have integrated uncertainty and neural networks [22,23].

In this paper, our main contributions are two-fold:

1. An unsupervised depth estimation network based on uncertainty is proposed to improve the problem of low prediction depth accuracy in monocular depth estimation. This method of uncertainty learning solves the problem in which the convolutional neural network currently used for monocular depth estimation has a strong expressive ability but cannot evaluate the reliability of the output result. By modeling the uncertainty, the confidence of the estimated depth can be predicted while the model prediction accuracy is improved and the uncertainty of the output result is quantified.
2. Retinex lighting theory is used to construct the photometric loss function to solve the interference problem caused by dynamic objects in the scene.

## 2. Materials and Methods

**Figure 1:**
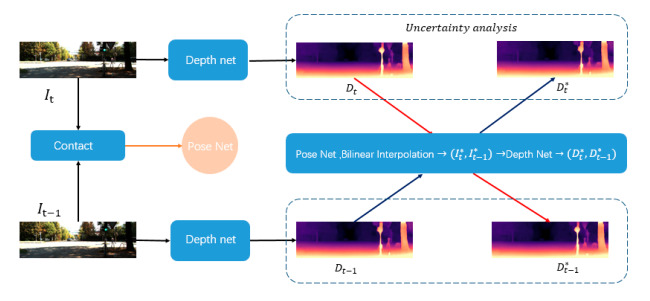
Monocular depth estimation network structure (all depth maps in the figure have pixel-level depth, which is absolute depth.)

Given two consecutive frames $I_{t}$ and $I_{t-1}$ sampled from an unlabeled video, we first estimate their depth maps $D_{t}$ and $D_{t-1}$ using the depth network, and then predict the relative 6D camera pose $P_{ab}$ between them using the PoseNet network. With the predicted depth map $D_{t}$ and the relative camera pose $P_{ab}$, we synthesize $I_{t}^{*}$ by warping $I_{t-1}$, where differentiable bilinear interpolation [24] is used as in [14]. Similarly, we obtain the image $I_{t-1}^{*}$. Finally, we input ($I_{t}^{*},I_{t-1}^{*}$) into the DepthNet to obtain $(D_{t}^{*},D_{t-1}^{*}$). We construct the loss function $L_{U}$ between $\left(D_{t},D_{t}^{*}\right)$ and $\left(D_{t-1},D_{t-1}^{*}\right)$ using uncertainty analysis. The structure of the network is shown in Figure 1.

The total loss function of the target network is:

(2) $$L\;=\;L_{R}+L_{s}+L_{U},$$

where $L_{R}$ represents the photometric loss, $L_{s}$ represents the loss of smoothness, and $L_{U}$ represents the uncertainty of the neural network.

### 2.1. Photometric Loss 

**Figure 2:**
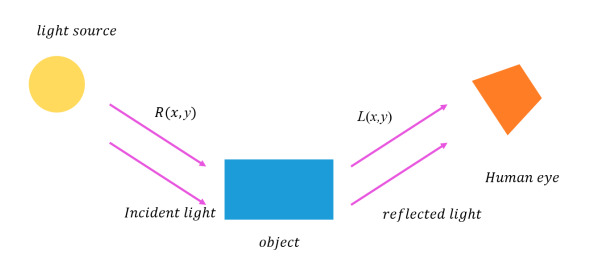
Retinex algorithm light decomposition diagram.

The basic theory of the Retinex algorithm is shown in Figure 2. $R\left(x,y\right)$ is incident light and $L\left(x,y\right)$ is reflected light. The incident light directly determines the dynamic range that the pixels in the image can reach, and the reflected light represents the image of the reflective nature of the object.

The change in the moving object directly affects the reflected light of $L\left(x,y\right)$ but does not affect the incident light of $R\left(x,y\right)$. Therefore, the network can be supervised from the $R\left(x,y\right)$ direction as a loss function to avoid the interference problem of dynamic objects.

According to the basic theory of the Retinex algorithm, the expression is as follows:

(3) $$I\left(x,y\right)\;=\;R\left(x,y\right)\times L\left(x,y\right).$$

The single-scale Retinex algorithm is often used for image enhancement. We apply it here to the establishment of the monocular depth estimation loss function. The main principle of the single-scale Retinex algorithm is convolving the three channels of the image with the center surround function. The image after the convolution operation is regarded as an estimate of the illumination component of the original image.

The process of using a low-pass filter to solve the incident component through a convolution operation can be expressed as:

(4) $$L\left(x,y\right)\;=\;I\left(x,y\right)*G\left(x,y\right).$$

From a mathematical perspective, solving $R\left(x,y\right)$ is a singular problem that can only be calculated by approximate estimation using mathematical methods. Assuming that the illumination image is estimated as a spatially smooth image, the incident light $R\left(x,y\right)$ can be obtained according to the single-scale Retinex algorithm:

(5) $$r_{i}\left(x,y\right)\;=\;log\left(R_{i}\left(x,y\right)\right)\;=\;log\left(\frac{I_{i}\left(x,y\right)}{L_{i}\left(x,y\right)}\right)\;=\;log\left(I_{i}\left(x,y\right)\right)-log\left(I_{i}\left(x,y\right)*G\left(x,y\right)\right)$$

where i represents the color channel, $R_{i}\left(x,y\right)$ represents the pixel value of the reflection image of the i color channel, $I_{i}\left(x,y\right)$ represents the pixel value of the original image $I\left(x,y\right)$ of the i color channel, * represents the convolution operation, and $G\left(x,y\right)$ represents the Gaussian surround function:

(6) $$G\left(x,y\right)\;=\;\frac{1}{2\pi \sigma^{2}}\exp\left(-\frac{x^{2}+y^{2}}{2\sigma^{2}}\right)$$

where σ represents the standard deviation in the Gaussian function, which is called the scale function here. The size of the standard deviation greatly affects the Retinex algorithm.

In summary, the photometric loss function can be transformed from Equations (7) and (8):

(7) $$L_{r}\;=\;||I_{t}-I_{t}^{*}{\left|\right|}_{1},$$

(8) $$L_{R}\;=\;\frac{1}{N}\underset{N}{\sum }||r_{i}{\left(x,y\right)}_{t}-r_{i}{\left(x,y\right)}_{t}^{*}||,$$

where *N* represents pixels in the image.

### 2.2. Smoothness Loss

Before regularizing the estimated depth map of the existing work, the smoothness loss needs to be added. We adopt the edge-aware smoothness loss used in [24], which is formulated as:

(9) $$L_{S}\;=\;\underset{N}{\sum }{\left(exp\left(-\nabla I_{t}\right)\times \nabla D_{t}\right)}^{2},$$

where ∇ is the first derivative along spatial directions, which ensures that smoothness is guided by the edge of images.

### 2.3. Uncertainty Analysis

The uncertainty of neural networks is generally divided into two categories: model uncertainty and random uncertainty. Model uncertainty mainly refers to the uncertainty of model parameters. When there are multiple models with good results, the final model parameters need to be selected from them. When the amount of input data is large enough, the model uncertainty is very low. In this paper, the training data were large enough, so the model uncertainty was not considered.

Sensor noise and motion noise may cause the observation data to be inaccurate, resulting in random uncertainty. These observation noises cannot be eliminated by large-scale data training. We assume that the data have a Gaussian distribution when modeling random uncertainties, and the likelihood function is shown in Equation (10).

(10) $$p\left(D|D^{*}\right)\;=\;N\left(D^{*},\sigma^{2}\right),$$

where D represents the depth observation data, $D^{*}$ represents the depth of the model output, and $\sigma^{2}$ represents the noise variance.

According to Equation (10), we take the logarithm of both sides of the equation and solve the negative log likelihood function:

(11) $$log\left(p\left(D|D^{*}\right)\right)\;=\;log\left(N\left(D^{*},\sigma^{2}\right)\right)\;=\;log\left(\frac{1}{\sqrt{2\pi }\sigma }exp\left(-\frac{{\left(D-D^{*}\right)}^{2}}{2\sigma^{2}}\right)\right),$$

(12) $$logp\left(D|D^{*}\right)\;=\;-\left(\frac{1}{2}log2\pi +\frac{1}{2}log\sigma^{2}+\frac{1}{2\sigma^{2}}{\left(D-D^{*}\right)}^{2}\right).$$

The random uncertainty of heteroscedasticity assumes that the noise variance is variable under different inputs. For example, uncertainties such as the edges of objects and distant scenes are usually higher, while other positions are more reliable. Therefore, the objective function of learning is as follows:

(13) $$L_{u}\;=\;\frac{1}{N}\overset{N}{\underset{i}{\sum }}(\frac{1}{2\sigma_{i}^{2}}||D_{t}-D_{t}^{*}{\left|\right|}_{2}^{2}+\frac{1}{2}log\sigma_{i}^{2}),$$

where *N* represents the number of pixels, $\left(D_{t},D_{t}^{*}\right)$ represents the depth value of the depth map, and $\sigma_{i}^{2}$ represents the variance output at the end of the network.

Depth estimation is a regression task. The most common loss functions for regression task optimization include the L2 loss function and the L1 loss function. The square operation makes the L2 loss function sensitive to outliers and it has a good optimization effect for large prediction errors, but has poor ability to further optimize for small prediction errors. The L1 loss function has a better optimization effect for smaller prediction errors, whereas the optimization effect for large prediction errors is general. The L1 loss function is slightly better in actual training. The uncertainty loss function proposed in this paper combines L1 loss and heteroscedastic random uncertainty in neural networks. In addition, the linear growth rate of the L1 loss makes it insensitive to loud noises, thus inhibiting adverse effects. 

The objective function of uncertainty can be expressed as Equation (14):

(14) $$L_{U}\;=\;\frac{1}{N}\overset{N}{\underset{i}{\sum }}(\frac{1}{2\sigma_{i}^{2}}||D_{t}-D_{t}^{*}{\left|\right|}_{1}+\frac{1}{2}log\sigma_{i}^{2}).$$

To avoid the denominator being zero and to ensure the loss function has better numerical stability, the uncertainty loss function is transformed into:

(15) $$L_{U}\;=\;\frac{1}{N}\overset{N}{\underset{i}{\sum }}(exp\left(-W_{i}\right)||D_{t}-D_{t}^{*}{\left|\right|}_{1}+W_{i}),$$

where $\sigma_{i}^{2}$ still represents the variance output at the end of the network, $W_{i}$ represents the value $log\sigma_{i}^{2}$, and i represents the index value.

## 3. Results

### 3.1. Experimental Environment

The software environment: Ubuntu 64-bit operating system, NVIDIA CUDA 9.1, NVIDIA CUDNN 7.1 and Python 3.7.0.

Hardware environment: Intel(R) Core (TM) i7-7700 CPU@3.60GHz processor, Kingston 32 GB memory, and NVIDIA GeForce GTX 1080Ti GPU, 11 GB.

### 3.2. Network Architecture

For the depth network, we experimented with DispNet [14], which takes a single RGB image as input and outputs a depth map. For the PoseNet network, we used a network without a mask prediction branch [14]. Using the total loss function proposed in this paper to train the network obtained a relatively ideal result. 

### 3.3. Evaluation Index

To objectively evaluate the proposed monocular depth estimation model, this paper uses the following five evaluation criteria to quantify the model:

Average relative error (Rel):

(16) $$\frac{1}{N}\sum_{i=1}^{N}\frac{\left|d_{gt}-d_{p}\right|}{d_{gt}}.$$

Root mean squared error (RMSE):

(17) $$\sqrt{\frac{1}{N}\sum_{i=1}^{N}{\left(d_{gt}-d_{p}\right)}^{2}}.$$

Average log10 error (log10):

(18) $$\frac{1}{N}\sum_{i=1}^{N}\left|\log_{10}d_{gt}-\log_{10}d_{p}\right|.$$

Accuracy with threshold thr:

(19) $$\mathrm{Percentage}\;(\%)\;\mathrm{of}\;s.t:\max\left(\frac{d_{gt}}{d_{p}},\frac{d_{p}}{d_{gt}}\right)\;=\;\delta <\mathrm{thr},$$

where $d_{gt}$ and $d_{p}$ are the ground-truth and predicted depths of pixels, respectively, and N is the total number of pixels in all the evaluated images.

### 3.4. Comparisons with the State-of-the-Art Methods

**Figure 3:**
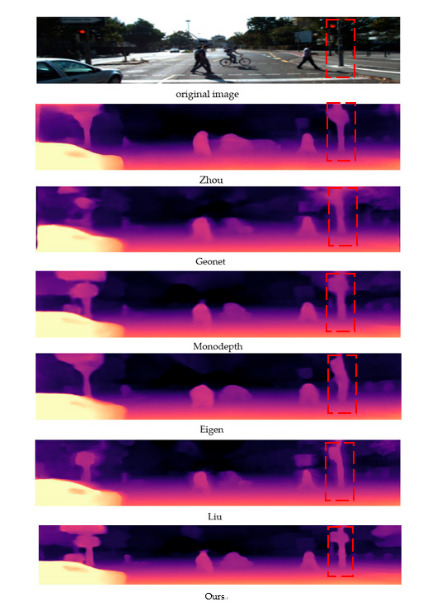
Comparison with current mainstream algorithms. The red dotted frame shows that the algorithm in this paper does not have pixels pulled around the relatively moving objects.

We evaluate the evaluation model on the KITTI dataset [25]. Figure 3 shows the results obtained, showing that the pixels around the moving object are not excessively deviated. The depth of pixels around moving objects is also not blurred. Table 1 provides the comparison between the results of this paper and other algorithms.

The experimental results showed that the algorithm proposed in this paper is as effective as the state-of-the-art algorithms. Our algorithm is slightly inferior to [16] in terms of SqRel and RNSlog. Reference [16] used a combination of supervised and unsupervised methods, using true depth labels. It also shows that the unsupervised learning method in this paper can achieve the accuracy of supervised learning. To better prove the effectiveness of the proposed method, we performed an ablation study, as described in Section 3.5.

### 3.5. Ablation Study

In this section, we verify the contributions of two innovations in this paper: luminosity loss and uncertainty analysis. We used the DispNet network for ablation study. The image resolution input in Table 2 is 416 × 128, and the image resolution input in Table 3 is 832 × 256. Among them, the methods are: ’Basic’, ’Basic + Retinex’, ’Basic + Uncertainty’, and ‘Basic + Retinex + Uncertainty’. The black bold in the Table 2 and Table 3 indicate the best results. The result clearly showed the overall improvement of the monocular depth estimation using our proposed scheme.

When the basic network part was optimized with Retinex, the error parameters of AbsRel, SqRel and RMS significantly reduced. We think that this occurred due to the reduction in the error rate of the proposed algorithm in the small part around the object, which also serves the purpose of constructing loss function $L_{R}$. After adding uncertainty analysis, the overall accuracy of monocular depth estimation increased, and the error rate decreased. This illustrated the importance of improving model prediction accuracy through modeling uncertainty.

## 4. Discussion

**Figure 4:**
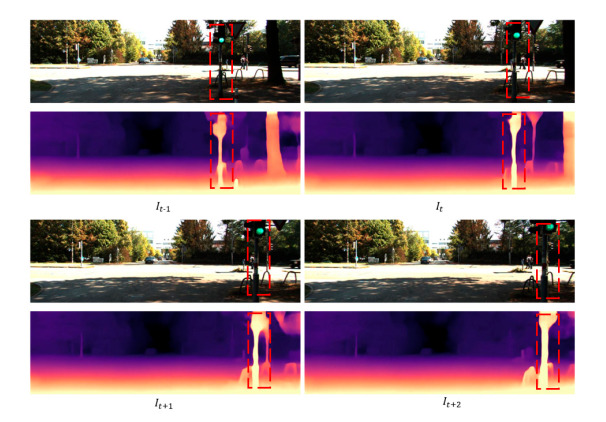
The result graph of the algorithm of the two adjacent frames.

Unlike the general regression task loss function, the uncertainty loss function proposed in this paper can not only estimate the depth, but also obtain the confidence of the estimated depth through the predicted variance. The smaller the noise variance, the closer the predicted depth to the real depth; the larger the noise variance, the higher the deviation between the predicted depth and the real depth. Figure 3 shows a detailed comparison of the mainstream algorithms and the algorithms in this article in recent years. According to Figure 4, there is no fuzzy pulling around the depth estimation objects of two adjacent frames, indicating that the proposed method is effective in solving the monocular depth estimation problem of moving objects. The pulling phenomenon around the moving object is improved and the network is monitored with uncertainty analysis. As can be seen from Table 1, compared with other algorithms in terms of accuracy, there is room for improvement.

## 5. Conclusions

This paper proposed a method of monocular depth estimation based on uncertainty and a method of optical flow loss function based on the Retinex algorithm as a supervised network. The proposed method solves the problem of pulling around pixels due to the presence of moving objects. State-of-the-art performance is achieved on the KITTI dataset. In future work, we will focus on the effectiveness of unsupervised depth estimation in more complex scenarios.

## Figures and Tables

**Table 1:** Objective analysis (The black bold in the table indicates the best result).

Method	AbsRel	SqRel	RMSE	RMSlog	<1.25	<1.25^2^	<1.25^3^
Eigen, D. et al. [2]	0.203	1.548	6.307	0.282	0.702	0.890	0.958
Liu et al. [26]	0.202	1.614	6.523	0.275	0.678	0.895	0.965
Garg et al. [19]	0.152	1.226	5.849	0.246	0.784	0.921	0.967
Kuznietsov et al. [21]	0.113	**0.741**	4.621	**0.189**	0.862	0.960	0.986
Godard et al. [23]	0.148	1.344	5.927	0.247	0.803	0.922	0.964
Zhan et al. [18]	0.144	1.391	5.869	0.241	0.803	0.928	0.969
Zhou et al. [14]	0.208	1.768	6.856	0.283	0.678	0.885	0.957
Mahjourian et al. [16]	0.163	1.240	6.220	0.250	0.762	0.916	0.968
Wang et al. [27]	0.151	1.257	5.583	0.228	0.810	0.936	0.974
Geonet et al. [15]	0.155	1.296	5.587	0.233	0.806	0.933	0.973
DF-Net [28]	0.150	1.124	5.507	0.223	0.806	0.933	0.973
CC [29]	0.140	1.070	5.326	0.217	0.826	0.941	**0.975**
Ours	**0.112**	0.792	**4.526**	0.191	**0.843**	**0.965**	0.967

**Table 2:** Ablation study (Input image resolution: 416 × 128).

Method	AbsRel	SqRel	RMSE	RMSlog	<1.25	<1.25^2^	<1.25^3^
Basic	0.161	1.225	5.765	0.237	0.780	0.927	0.972
Basic + Retinex	0.132	0.905	4.689	0.196	0.791	0.935	0.974
Basic + Uncertainty	0.152	0.836	4.634	0.199	0.801	0.942	0.965
Basic + Retinex + Uncertainty	**0.112**	**0.792**	**4.526**	**0.191**	**0.843**	**0.965**	**0.967**

**Table 3:** Ablation study (Input image resolution: 832 × 256).

Method	AbsRel	SqRel	RMSE	RMSlog	<1.25	<1.25^2^	<1.25^3^
Basic	0.151	1.154	5.716	0.232	0.798	0.930	0.972
Basic + Retinex	0.129	1.023	4.785	0.196	0.802	0.923	0.974
Basic + Uncertainty	0.145	0.866	4.854	0.201	0.800	0.915	0.975
Basic + Retinex + Uncertainty	**0.127**	**0.892**	**4.625**	**0.189**	**0.822**	**0.939**	**0.977**
